# Phylogeny, Diet, and Cranial Integration in Australodelphian Marsupials

**DOI:** 10.1371/journal.pone.0000995

**Published:** 2007-10-03

**Authors:** Anjali Goswami

**Affiliations:** 1 Committee on Evolutionary Biology, University of Chicago, Chicago, Illinois, United States of America; 2 Department of Geology, The Field Museum, Chicago, Illinois, United States of America; Ecole Normale Supérieure de Lyon, France

## Abstract

Studies of morphological integration provide valuable information on the correlated evolution of traits and its relationship to long-term patterns of morphological evolution. Thus far, studies of morphological integration in mammals have focused on placentals and have demonstrated that similarity in integration is broadly correlated with phylogenetic distance and dietary similarity. Detailed studies have also demonstrated a significant correlation between developmental relationships among structures and adult morphological integration. However, these studies have not yet been applied to marsupial taxa, which differ greatly from placentals in reproductive strategy and cranial development and could provide the diversity necessary to assess the relationships among phylogeny, ecology, development, and cranial integration. This study presents analyses of morphological integration in 20 species of australodelphian marsupials, and shows that phylogeny is significantly correlated with similarity of morphological integration in most clades. Size-related correlations have a significant affect on results, particularly in Peramelia, which shows a striking decrease in similarity of integration among species when size is removed. Diet is not significantly correlated with similarity of integration in any marsupial clade. These results show that marsupials differ markedly from placental mammals in the relationships of cranial integration, phylogeny, and diet, which may be related to the accelerated development of the masticatory apparatus in marsupials.

## Introduction

The correlated evolution of traits is a fundamental issue in evolutionary biology, with great importance for understanding morphological evolution and the generation of morphological diversity [1]–[3]. Morphological integration is the study of trait associations, measured through statistical analysis of patterns of trait covariation or correlation. Integration of functionally or developmentally-related traits can influence morphological evolution in many ways, from constraining the variability of individual traits to facilitating transformations of functional sets [1]–[10]. Yet, despite this importance to morphological evolution, trait integration has been overlooked in most morphological analyses.

Most studies of morphological integration focus on microevolutionary hypotheses , documenting the relationships among development, genetics and phenotypic integration, usually in single species [for recent reviews see 2,3,10,11]. The few comparative studies conducted have focused on placental mammals [11]–[17]. A single study has included marsupials and monotremes, as well as placentals, and has shown a high degree of conservation of cranial integration across therian mammals [10]. However, differences in patterns of trait integration do exist among therian taxa, and these differences may be ascribed to several factors. Phylogeny and ecology are of particular interest, as they have been correlated with similarity of cranial integration in the placental clades Primates [13], [15], [16] and Carnivora [11].

Because placentals and marsupials differ greatly in the timing of cranial bone ossification [18]–[22], and because developmental timing has often been considered to be a major influence on integration [14], [23]–[29], examination of integration in marsupials will provide an important comparison to the extensive studies of placental mammals. In this paper, I assess the relationship between evolutionary history, ecology, and cranial integration in australodelphian marsupials. Specifically, I test the hypothesis that similarity in cranial integration in marsupials is correlated with phylogeny relationship and dietary similarity.

A plausible null hypothesis is that evolutionary history (phylogeny) is correlated with similarity in patterns of morphological integration. Of the placental clades studied, however, only a few support this hypothesis [11], [12]. Other clades display only a weak correlation between phylogeny and cranial integration [15], [16], [30], while some clades show stronger correlations between diet and morphological integration [11], [16]. Diet strongly influences tooth size and shape and jaw musculature, and thus overall skull morphology. Skulls must accommodate the functional demands of juvenile and adult food processing, and, if masticatory traits are functionally integrated, then similarities in diet may be reflected in similarity in morphological integration.

These results from previous studies demonstrate that a complex relationship exists between phylogenetic relatedness, integration, and ecology across placental mammals. In addition, as suggested by Steppan [31], disparate microevolutionary and macroevolutionary processes may manipulate morphological integration. While it is clear that evolutionary history is related to morphological integration to some extent, it is not understood how general this relationship is, nor how significant patterns of integration are in morphological evolution.

As noted above, examination of morphological integration in marsupials is particularly important, because of the striking differences in the timing of cranial bone development between marsupials and placentals. Ossification of the anterior masticatory apparatus (premaxilla, maxilla, and dentary) is accelerated in marsupials relative to placentals. This heterochronic shift is related to the unique marsupial reproductive pattern in which neonates are birthed after a short gestation period and complete their early development attached to the teat [22]. If this early ossification and use of the masticatory apparatus influences the developmental integration of those bones, it may also affect potential functional integration related to adult diet. Therefore, this study of morphological integration in marsupials will provide the data to assess, in comparison with placental mammals, how heterochrony may be influencing morphological integration. Comparisons among marsupial and placental mammalian clades thus provides an opportunity to isolate three of the factors (phylogeny, ecology, and development) that have often been invoked as influences on morphological integration and morphological evolution.

## Materials and Methods

### Data collection

**Figure 1:**
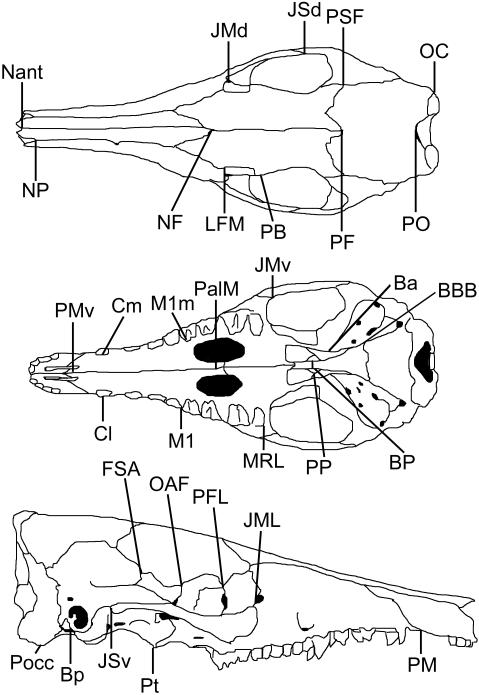
Cranial landmarks, shown on *Echymipera kalubu.* Symmetrical landmarks are shown on one side only.

Cranial landmarks were captured using an Immersion Microscribe G2×3-D digitizer. Fifty-seven landmarks were collected across the skull, emphasizing points of certain homology across taxa, such as tripartite sutures. In addition, landmarks corresponding to those in earlier studies also were used, to permit direct comparison with previous results. Landmarks are listed in Table 1 and illustrated in Figure 1 (symmetrical landmarks are displayed on one side only).

**Table 1:** Cranial landmarks

Symbol	Landmark
**PMv**	**Premaxilla–Maxilla ventral suture**
PM	Premaxilla–Maxilla anterior suture
**Nant**	**Nasal–anterior extreme**
NP	Nasal–Premaxilla–anterior suture
Cl	Canine–lateral extreme
Cm	Canine–mesial extreme
M1	Anterior lateral M1
MRL	Posterior lateral M2
M1m	Anterior mesial M1
**PalM**	**Palatine–Maxilla ventral suture**
JMv	Jugal–Maxilla ventral suture
JMd	Jugal–Maxilla dorsal suture
**NF**	**Nasal–Frontal suture**
JML	Jugal–Maxilla–Lacrimal suture
LFM	Lacrimal–Frontal–Maxilla suture
PB	Postorbital process of the frontal
Ba	Bulla–anterior extreme
Pt	Pterygoid–posterior extreme
PFL	Palatine–Frontal–Lacrimal suture
OAF	Orbitosphenoid–Alisphenoid–Frontal suture
BP	Basisphenoid–Presphenoid suture
PP	Presphenoid–Palatine suture
JSv	Jugal–Squamosal ventral suture
JSd	Jugal–Squamosal dorsal suture
Bp	Bulla–posterior extreme
POcc	Paraoccipital process
OC	Occipital condyle–lateral extreme
BBB	Basioccipital-Basisphenoid-Bulla suture
**PF**	**Parietal–Frontal suture**
**PO**	**Parietal–Occipital suture**
PSA	Parietal–Squamosal–Alisphenoid suture
PFA	Parietal–Frontal–Alisphenoid suture

Shown in Figure 1. Midline traits are marked in bold.

Twenty species of australodelphian marsupials were included in this analysis, spanning Dasyuromorphia, Peramelia, and Diprotodontia (Appendix S1). Taxa were chosen due to their morphological convergence with placental groups in which cranial integration has been studied (Phalangeridae on Primates; Dasyuromorphia on Carnivora), their relationship to fossil taxa, not included in this study, that are convergent with placental taxa (Vombatidae to Thylacoleonidae, which are convergent with placental carnivorans), or their unique developmental patterns (Peramelia). As this study focuses on more inclusive clades, only a few congeneric species are included to provide a broad range of phylogenetic relationships. Dasyuridae (native ‘cats’ and marsupial ‘mice’) is better sampled than others, due to its taxonomic and ecological diversity and the availability of sufficient specimens in many museum collections, and will be used to examine within-family patterns.

Data were collected from 13 to 16 adult specimens per species, for a total of 327 specimens from 20 species, and male and female specimens are as equally represented as possible (Appendix S1). While higher specimen numbers is preferred, many of the taxa of interest are rare, and ten of the largest international collections were visited to attain this sample. A series of rarefaction and bootstrap analyses were conducted to determine that matrix correlation analysis and pairwise trait correlations were stable at these sample sizes [11]. Furthermore, rarefaction analyses also show that matrix correlations between two species decrease with reduced sample size. Therefore, the effect of lower sample sizes, if any, will be to reject real similarity in patterns of integration and to reduce the significance of results, rather than to create false similarity and increase significances. This methodology is thus more conservative and would impact all analyses for phylogeny and diet equally.

### Data analysis

Analytical methods follow previous studies [10], [11], and a brief review of methodology is provided here. Only landmarks from the midline (6) and right side of the skull (26) were used in analyses. Specimens were aligned with Procrustes analysis, using an algorithm written in Mathematica 5.0 (Wolfram Research Inc., Champaign, IL). Scaling, a common Procrustes procedure, was not applied to specimens, to reduce the effect of inducing covariances through Procrustes fitting. Pearson product-moment dot covariances were calculated for individual species in Mathematica 5.0. For some analyses, the first eigenvector, which mainly reflects size, was removed from the covariance matrix prior to matrix correlation analysis. Comparisons among results including and excluding the first eigenvector allow us to estimate the role of size in morphological integration, as analyses of these data have shown that the first eigenvector is a proxy for body size (although size still influences the remaining eigenvectors). Trait variance-covariance matrices were converted to trait correlation matrices by dividing covariances by respective variances. These steps produce a 32×32 trait correlation matrix for each species.

Matrix correlation analysis was employed to assess similarity in patterns of morphological integration [11], [12], [16], [30], [32]. Trait correlation matrices for each species were compared to that of every other species, using matrix correlation analysis. The matrix correlations between species were used to build the matrix of similarity of integration (hereafter, MSI), which consists of pairwise matrix correlations (Appendix S2). MSI was used to assess the association of phylogenetic relatedness or dietary similarity with similarity in cranial integration. Analyses were conducted at several phylogenetic levels and were restricted to clades with more than five species sampled (Appendix S1): Marsupialia; Dasyuromorphia; Peramelia; Diprotodontia; and Dasyuridae.

### Phylogeny

To test the relationship between MSI and phylogenetic relatedness, multiple phylogenetic similarity matrices were constructed for all of the taxa examined, using recently published phylogenetic hypotheses [33]–[43]. Recent phylogenetic hypotheses incorporating the taxa examined in this study differed in the relative placements of the three orders examined in this study. Some studies placed Dasyuromorphia as basal to Peramelia+Diprotodontia [35], [36], some placed Peramelia as basal to Dasyuromorphia+Diprotodontia [37], and still others placed Dasyuromorphia and Peramelia as sister groups relative to Diprotodontia [38], [39].

**Figure 2:**
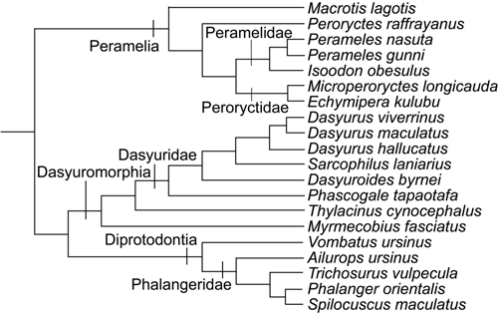
One phylogenetic hypothesis for taxa included in this study [37], [42]. Alternative phylogenies, with Peramelia basal and with different topologies within Peramelia, were also tested.

There is also a lack of consensus on the relationships within Peramelia. Groves and Flannery [41] recognised two families, Peramelidae (*Perameles*, *Isoodon*, and *Macrotis*) and Peroryctidae (*Peroryctes*, *Microperoryctes*, and *Echymipera*). Szalay [40] placed *Macrotis* as the nearest outgroup to the rest of the peramelians included in this study, while Westerman et al. [42] also placed *Peroryctes* outside the remaining peramelians in this study. Each of these competing phylogenetic hypotheses for Marsupialia and for Peramelia was analysed separately to test the relationship between phylogeny and similarity of morphological integration (Fig 2).

For each topology, the patristic distance between each pair of species was calculated. Because a similarity matrix is required for comparison, each patristic distance was subtracted from the maximum value among species (those related only as australodelphian marsupials)+1, such that the most distantly-related species have a value of one and sister taxa have the maximum value. Matrix correlation analysis was used to test the correlation of various phylogenetic distance matrices with MSI. Mantel's test is used to determine the significance of the matrix correlation. Mantel's test randomly reorders the rows and columns of one of the two correlation matrices being compared and recalculates the matrix correlation between the two matrices [44]. This operation was repeated 10,000 times, providing a random distribution of matrix correlations with which to assess the significance of the observed matrix correlation.

An alternative analysis of phylogenetic relationship also was employed. Pairwise similarity of morphological-integration values were averaged for taxa related at various taxonomic levels (single pairs analysis [32]). For example, the matrix correlations between all pairs of species that are related at the genus level are averaged, versus all pairs that are related at the family level but not at the genus level, etc. This analysis was conducted among all species and within individual orders for four taxonomic levels of relationship: infraclass, order, family, and genus. If phylogenetic relatedness is correlated with similarity in morphological integration, average pairwise MSI values should decrease from the generic to the infraclass level. Analysis of variance (ANOVA) was conducted to determine if there are significant differences in similarity of morphological integration across taxonomic ranks.

### Diet

To test the correlation between MSI and similarity in diet, a dietary similarity matrix was constructed among all taxa, based on the proportion of shared diet between species. This analysis followed the methodology of Marroig and Cheverud [16] for quantifying similarity in diet based on the proportion of shared dietary types. Each species was categorised by the approximate percentage of vertebrates, invertebrates/insects, fruits, and leaves in its diet (Appendix S1). Dietary information was taken from existing literature, using approximated contributions of each category to a species' total diet [45], [46].

Dietary similarity between two species was calculated as a sum across the four categories, where each category had a value comprised of the square root of the product of each species' percentage for that particular dietary type [see 11]. This process was repeated for each pair of taxa, resulting in a matrix of dietary similarity. The dietary similarity matrix (hereafter DSM) was then compared to MSI using matrix correlation analysis with Mantel's test for significance.

Phylogenetic relatedness has the potential to complicate the analysis of diet, due to the possibility that more closely-related taxa are more similar in diet because of common ancestry alone. To test for the possible influence of phylogeny, the dietary-similarity matrix was compared to the phylogenetic-distance matrix, using matrix correlation analysis with a Mantel's test for significance. The dietary-similarity matrix was significantly correlated with the node-based phylogenetic-distance matrices using basal Peramelia (r = 0.55, p<0.001), basal Dasyuromorphia (r = 0.56, p<0.001), and Dasyuromorphia+Peramelia (r = 0.64, p<0.001). Because diet is significantly correlated with phylogeny, the dietary-similarity matrix was regressed against the phylogenetic-distance matrix to isolate diet from phylogeny. The dietary similarity residual matrix (hereafter DSRM) was compared to the original MSI, using matrix correlation analysis with Mantel's test for significance.

## Results

### Phylogeny

**Figure 3:**
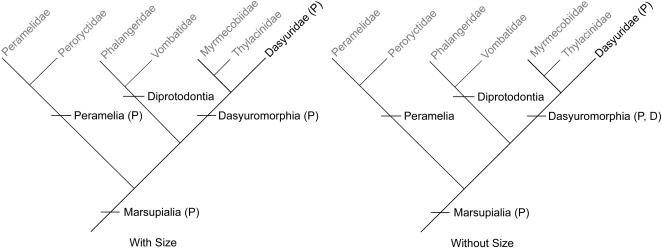
One phylogenetic hypothesis for taxa included in this study [33], [34], [37], [42], [43] showing taxa with significant or marginally significant correlations between similarity in morphological integration and phylogeny (P) or diet (D). Taxa labeled in gray do not have enough species sampled in this study for statistical analysis.

Across all australodelphian marsupials, there was a significant correlation with phylogeny using all topologies (Fig. 3, Table 2). This correlation was statistically robust to the inclusion or exclusion of size. Across the smaller clades examined in this study, however, only Dasyuromorphia and Dasyuridae showed a significant correlation with phylogeny. Peramelia showed a marginally significant correlation with phylogeny in all three topologies when size was included, but in none when size was removed. Diprotodontia, represented by Vombatidae and Phalangeridae, did not show a significant correlation with phylogeny in the analyses with or without size.

**Table 2:** Phylogenetic relationship and cranial integration.

Group	Species	R with size	R without size
Marsupialia–Peramelia basal	20	.55**	.41**
Marsupialia–Dasyuromorphia basal	20	.45**	.40*
Marsupialia–Peramelia+Dasyuromorphia	20	.55**	.42**
Dasyuromorphia	8	.80**	.90**
Dasyuridae	5	.86**	.90**
Peramelia [41]	7	.73*	.43
Peramelia [40]	7	.71*	.43
Peramelia [42]	7	.71*	.45
Diprotodontia	5	.86	.61

Results from matrix correlation analysis of phylogenetic distance matrix and matrix of similarity in morphological integration. Asterisks indicate significance at the p = 0.05 level (**) or the p = .1 level (*).

**Figure 4:**
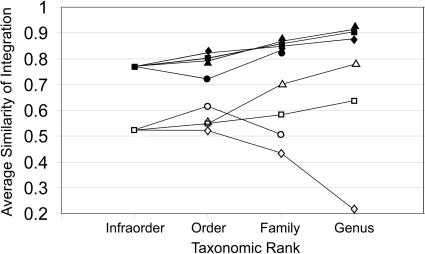
Average matrix correlation between species related at increasingly disparate taxonomic levels for all australodelphian marsupials (▪), Dasyuromorphia (▴), Diprotodontia (•), and Peramelia (♦), with (closed symbols) and without size (open symbols).

Single pairs analysis was also conducted for each clade (Fig. 4). When size was included, average similarity of integration increased significantly (ANOVA, p<0.001) from species related only as australodelphian marsupials (0.77) to those in the same order (0.81), same family (0.86), and same genus (0.90). There were also significant increases in average similarity of integration with closer phylogenetic relationship within Dasyuromorphia (p<0.001), Peramelia (p<0.001), and Diprotodontia (p = 0.01). When size was removed, there was no significant relationship between taxonomic rank and similarity in integration across all australodelphian marsupials, although average similarity of integration slightly increased from infraclass (0.53), to order (0.55) to family (0.60), and to genus (0.61). Without size, Dasyuromorphia exhibited a significant similarity increase with phylogenetic relationship (p = .001), while Peramelia and Diprotodontia showed nonsignificant decreases in similarity of integration with phylogenetic relationship when size was removed.

### Diet

Neither DSM nor DSRM were significantly correlated with similarity in morphological integration in any of the clades examined in this study (Fig. 3, Table 3). Results did not differ significantly among the three phylogenetic hypotheses used to calculate DSRM, and results are presented solely for the most recent phylogeny [37]. Across Dasyuromorphia, there was a marginally significant correlation with DSM when size was excluded. In other analyses, size-related correlations do not significantly affect results.

**Table 3:** Dietary similarity and cranial integration.

Group	DSM		DSRM	
	with size	without size	With size	without size
Marsupialia	.25	.24	−.06	.03
Dasyuromorphia	.61	.60*	.21	.13
Dasyuridae	.05	.12	.04	.11
Peramelia	.41	.40	.20	.25
Diprotodontia	.42	.28	.11	.09

Results of matrix correlation analysis of dietary similarity matrix (DSM), dietary similarity residual matrix (DSRM), and matrix of similarity in morphological integration (MSI). Asterisks indicate significance at the p = 0.05 level (**) or the p = .1 level (*).

## Discussion

Within placental mammals, morphological integration has been analysed comparatively in Primates [12]–[17], [47], [48] and Carnivora [11]. Within both of these placental clades, correlation with phylogeny and diet varies, with some subclades showing significant correlations with phylogeny, some with diet, and others with neither factor. In both clades, size-related correlations significantly affect the relationship between phylogeny and cranial integration, but have little effect on the relationship between diet and cranial integration. Size is a evolutionary important factor, and size is often the dominant factor influencing trait variances and covariances [49] . While size remains an important factor influencing morphological integration in marsupials, the relationships among phylogeny, diet, and similarity of integration are quite different than those observed in placentals.

Matrix correlation analysis and single pairs analysis produced consistent results in most analyses. Both support a significant relationship between phylogeny and similarity of integration across australodelphian marsupials. These three orders are quite morphologically distinct and diverged 40–50 million years ago [38]; therefore, this correlation between phylogenetic relationship and similarity in morphological integration is perhaps expected.

The three orders examined, however, display three different patterns with respect to phylogeny, size, and cranial integration. While Dasyuromorphia and Dasyuridae show significant correlations between phylogenetic distance and similarity of integration, both including and excluding size (Table 2), Diprotodontia only shows a correlation between phylogeny and integration in single pairs analysis, when size is included (Fig. 4). It should be noted that, because only phalangerids and a single vombatid were sampled, results for Diprotodontia may mainly reflect the lack of a phylogenetic signal within Phalangeridae, rather than in all diprotodontians.

Peramelia shows an intermediate pattern between Dasyuromorphia and Diprotodontia, with marginally significant correlations in matrix correlation analysis when size is included, but not when it is removed. All three phylogenetic hypotheses for Peramelia produced similar results. Interestingly, the differences in correlation values between analyses with and without size are greater in Peramelia than in other clades. Likewise, in single pairs analysis, Peramelia shows increased similarity of integration with phylogenetic relationship when size is included, but a negative correlation when size is removed. This result seems to be primarily influenced by a few taxa (e.g., *Peroryctes*) that show particularly low similarity of integration with other taxa, whether including or excluding size. However, *Perameles nasuta*, which shows comparatively high similarity of integration with other peramelians when size is included, displays the lowest similarity of integration values when size is removed, most notably with the congeneric species *Perameles gunnii* (0.22). As the congeneric peramelid species reflects only a single comparison, between *Perameles nasuta* and *Perameles gunnii*, greater sampling of congeneric species is necessary to determine if that low similarity of integration among species is a general characteristic of Peramelia. However, these results suggest that size-related correlations are a more significant factor within Peramelia than in the other marsupial orders considered in this study, even though they occupy a smaller range of size than either Dasyuromorphia or Diprotodontia [46].

These differences in the relative influence of phylogeny and of size on patterns of morphological integration are of potential importance to understanding macroevolutionary trends in morphological integration and differences in evolutionary patterns across large clades. These analyses demonstrate that size and phylogeny are correlated with similarity in patterns of integration, but with strikingly disparate influences in the examined clades. If trait correlations significantly influence morphological variation, then these patterns provide the diversity necessary to isolate and test the evolutionary consequences of different patterns of morphological integration with empirical data from real species.

In contrast to the results for phylogeny, no clade in this analysis shows a significant correlation between similarity of integration and diet (Table 3). Among australodelphian marsupial orders, only Dasyuromorphia shows a marginally significant correlation between DSM and similarity of integration, and only when size is removed from analysis. Because this relationship is not observed when dietary similarity is regressed against phylogeny (DSRM), it is probable that the marginally significant correlation between DSM and similarity of integration merely reflects the strong correlation between phylogeny and similarity of integration. While both phylogeny and diet are strongly correlated with similarity in integration in placental taxa, although often in different clades, this study suggests that only phylogeny plays a significant role in morphological integration in australodelphian marsupials.

Diet is expected to influence morphological integration by inducing the functional integration of traits required for mastication. The marsupial species examined in this study include a broad range of ecological and morphological diversity, including hypercarnivorous (*Thylacinus*), invertivorous (*Myrmecobius*), and folivorous (*Trichosurus*, *Vombatus*) taxa, to species with a variety of mixed diets (Appendix S1). Therefore, this result does not simply reflect a lack of dietary diversity in sampled taxa, although marsupials are often considered to be less diverse in morphology and ecology than placentals. This lack of diversity has often been attributed to the observation that, in marsupials, the ossification of bones associated with feeding (premaxilla, maxilla, dentary) has been accelerated to accommodate the early birth and suckling of marsupials, relative to placentals [18]–[20], [22], [50]. If developmental timing or developmental integration is a major influence on morphological integration, then the early ossification of these bones that are typically associated with mastication may overshadow any diet-specific functional integration.

It is important to note that the relationship between morphological integration and morphological evolution are poorly understood [10]. A recent study of cranial shape in carnivorous marsupials [51], primarily dasyuromorphians, showed a strong correlation between diet and cranial shape. As discussed above, Dasyuromorphia was the only clade in this study to show a marginally significant correlation between cranial integration and diet, perhaps suggesting some relationship between morphological integration and cranial shape. While there are many hypotheses on the potential influence of character integration on morphological evolution, these have yet to be explicitly tested. Dasyuromorphia may well provide an ideal system for future studies of morphological integration's evolutionary significance, as cranial shape, ecomorphology, and, with this study, cranial integration, are all well studied for this clade.

This comparative study of morphological integration in the australodelphian cranium demonstrates that a broad range of patterns exist in the relationships among phylogeny and similarity in integration, but that phylogeny is significantly correlated with similarity in integration in most clades. In contrast, while all examined placental orders exhibit some significant correlation between diet and similarity in integration, australodelphian marsupials do not show this relationship in any clade. These results support the finding of a previous study [11] that phylogeny is a primary factor influencing patterns of morphological integration in all large clades, while diet is a significant factor in only some clades. This study also suggests that the early ossification of the facial skeleton in marsupials may influence patterns of cranial integration and the relative importance of ecology in shaping morphological integration.

## Supporting Information

Appendix S1 Species list, specimen numbers. Dietary categories used in construction of the dietary similarity matrix are invertivore (I), frugivore (Fr), folivore (Fo), and carnivore (C). *The diet of Vombatus is primarily grasses and roots.
(0.06 MB DOC)

Appendix S2 Matrix of similarity of morphological integration. The lower triangle is the original MSI. The upper triangle is MSI without size.
(0.15 MB DOC)
