# Supplementary material for: Phylogeny, Diet, and Cranial Integration in Australodelphian Marsupials
Source: PLoS One. 2007 Oct 3;2(10):e995. doi: 10.1371/journal.pone.0000995 (PMC1994583; doi:10.1371/journal.pone.0000995)
Supplement: Appendix S1 — Species list, specimen numbers. Dietary categories used in construction of the dietary similarity matrix are invertivore (I), frugivore (Fr), folivore (Fo), and carnivore (C). *The diet of Vombatus is primarily grasses and roots. (0.06 MB DOC) [file pone.0000995.s001.doc]

Appendix S1. Species list, specimen numbers (N), and dietary information.

Dietary categories used in construction of the dietary similarity matrix are invertivore (I), frugivore (Fr), folivore (Fo), and carnivore (C). *The diet of Vombatus is primarily grasses and roots.

| **Family** | **Species** | **Common name** | **N** | **Diet** | | | |
| --- | --- | --- | --- | --- | --- | --- | --- |
|  |  |  |  | **I** | **Fr** | **Fo** | **C** |
| Dasyuridae | *Dasyurus hallucatus* | Northern quoll | 15 | 0.20 | 0.00 | 0.10 | 0.70 |
|  | *Dasyurus maculatus* | Spotted-tailed quoll | 14 | 0.10 | 0.00 | 0.00 | 0.90 |
|  | *Dasyurus viverrinus* | Eastern quoll | 16 | 0.70 | 0.10 | 0.00 | 0.20 |
|  | *Phascogale tapaotafa* | Brush-tailed phascogale | 15 | 0.40 | 0.00 | 0.00 | 0.60 |
|  | *Sarcophilus laniarus* | Tasmanian devil | 16 | 0.10 | 0.00 | 0.00 | 0.90 |
|  | *Dasyuroides byrnei* | Kowari | 15 | 0.50 | 0.00 | 0.00 | 0.50 |
| Myrmecobiidae | *Myrmecobius fasciatus* | Numbat | 14 | 1.00 | 0.00 | 0.00 | 0.00 |
| Thylacinidae | *Thylacinus cynocephalus* | Tasmanian wolf | 15 | 0.00 | 0.00 | 0.00 | 1.00 |
| Phalangeridae | *Trichosurus vulpecula* | Silver-gray  brushtail possum | 15 | 0.00 | 0.00 | 1.00 | 0.00 |
|  | *Phalanger orientalis* | Gray cuscus | 15 | 0.10 | 0.40 | 0.50 | 0.00 |
|  | *Ailurops ursinus* | Bear cuscus | 15 | 0.00 | 0.10 | 0.90 | 0.00 |
|  | *Spilocuscus maculatus* | Short-tailed  spotted cuscus | 15 | 0.00 | 0.10 | 0.80 | 0.10 |
| Vombatidae | *Vombatus ursinus* | Coarse-haired wombat | 15 | 0.00 | 0.00 | 1.00* | 0.00 |
| Peramelidae | *Perameles nasuta* | Long-nosed bandicoot | 15 | 0.80 | 0.10 | 0.00 | 0.10 |
|  | *Perameles gunnii* | Eastern barred bandicoot | 14 | 0.80 | 0.10 | 0.00 | 0.10 |
|  | *Isoodon obesulus* | Southern brown bandicoot | 15 | 0.60 | 0.20 | 0.20 | 0.00 |
|  | *Macrotis lagotis* | Greater bilby | 15 | 0.80 | 0.20 | 0.00 | 0.00 |
| Peroryctidae | *Echymipera kalubu* | Kalubu echymipera | 15 | 0.40 | 0.00 | 0.60 | 0.00 |
|  | *Microperoryctes longicauda* | Striped bandicoot | 15 | 0.00 | 0.00 | 0.00 | 0.00 |
|  | *Peroryctes raffrayanus* | Raffray’s bandicoot | 13 | 0.50 | 0.00 | 0.25 | 0.25 |
