# Supplementary material for: Phylogeny, Diet, and Cranial Integration in Australodelphian Marsupials
Source: PLoS One. 2007 Oct 3;2(10):e995. doi: 10.1371/journal.pone.0000995 (PMC1994583; doi:10.1371/journal.pone.0000995)
Supplement: Appendix S2 — Matrix of similarity of morphological integration. The lower triangle is the original MSI. The upper triangle is MSI without size. (0.15 MB DOC) [file pone.0000995.s002.doc]

Appendix S2. Matrix of similarity of morphological integration (MSI).

The lower triangle is the original MSI. The upper triangle is MSI without size.

|  | *D. hallucatus* | *D. maculatus* | *D. viverrinus* | *Phascogale* |
| --- | --- | --- | --- | --- |
| *D. hallucatus* | 1.000 | 0.708 | 0.763 | 0.685 |
| *D. maculatus* | 0.893 | 1.000 | 0.860 | 0.559 |
| *D. viverrinus* | 0.897 | 0.947 | 1.000 | 0.663 |
| *Phascogale* | 0.874 | 0.874 | 0.898 | 1.000 |
| *Sarcophilus* | 0.909 | 0.912 | 0.927 | 0.846 |
| *Dasyuroides* | 0.836 | 0.799 | 0.850 | 0.818 |
| *Myrmecobius* | 0.727 | 0.634 | 0.706 | 0.728 |
| *Thylacinus* | 0.875 | 0.891 | 0.949 | 0.868 |
| *Trichosurus* | 0.875 | 0.867 | 0.905 | 0.892 |
| *Phalanger* | 0.800 | 0.792 | 0.807 | 0.780 |
| *Ailurops* | 0.760 | 0.740 | 0.779 | 0.782 |
| *Spilocuscus* | 0.864 | 0.850 | 0.923 | 0.839 |
| *Vombatus* | 0.744 | 0.665 | 0.741 | 0.760 |
| *P. nasuta* | 0.760 | 0.779 | 0.880 | 0.828 |
| *P. gunnii* | 0.712 | 0.696 | 0.821 | 0.769 |
| *Isoodon* | 0.850 | 0.886 | 0.929 | 0.868 |
| *Macrotis* | 0.860 | 0.889 | 0.937 | 0.885 |
| *Echymipera* | 0.789 | 0.796 | 0.901 | 0.837 |
| *Microperoryctes* | 0.683 | 0.671 | 0.789 | 0.718 |
| *Peroryctes* | 0.675 | 0.673 | 0.783 | 0.724 |

|  | *Sarcophilus* | *Dasyuroides* | *Myrmecobius* | *Thylacinus* | *Trichosurus* |
| --- | --- | --- | --- | --- | --- |
|  |  |  |  |  |  |
| *D. hallucatus* | 0.756 | 0.731 | 0.482 | 0.696 | 0.353 |
| *D. maculatus* | 0.852 | 0.622 | 0.366 | 0.672 | 0.289 |
| *D. viverrinus* | 0.822 | 0.717 | 0.411 | 0.687 | 0.360 |
| *Phascogale* | 0.608 | 0.696 | 0.459 | 0.579 | 0.332 |
| *Sarcophilus* | 1.000 | 0.685 | 0.378 | 0.733 | 0.338 |
| *Dasyuroides* | 0.858 | 1.000 | 0.469 | 0.663 | 0.324 |
| *Myrmecobius* | 0.726 | 0.733 | 1.000 | 0.510 | 0.697 |
| *Thylacinus* | 0.904 | 0.850 | 0.725 | 1.000 | 0.519 |
| *Trichosurus* | 0.874 | 0.745 | 0.708 | 0.863 | 1.000 |
| *Phalanger* | 0.816 | 0.721 | 0.630 | 0.760 | 0.845 |
| *Ailurops* | 0.776 | 0.738 | 0.613 | 0.768 | 0.831 |
| *Spilocuscus* | 0.892 | 0.797 | 0.695 | 0.918 | 0.905 |
| *Vombatus* | 0.784 | 0.726 | 0.710 | 0.777 | 0.744 |
| *P. nasuta* | 0.816 | 0.812 | 0.708 | 0.851 | 0.803 |
| *P. gunnii* | 0.745 | 0.744 | 0.677 | 0.810 | 0.744 |
| *Isoodon* | 0.866 | 0.792 | 0.712 | 0.886 | 0.869 |
| *Macrotis* | 0.894 | 0.851 | 0.689 | 0.922 | 0.846 |
| *Echymipera* | 0.827 | 0.818 | 0.721 | 0.867 | 0.804 |
| *Microperoryctes* | 0.748 | 0.733 | 0.758 | 0.789 | 0.713 |
| *Peroryctes* | 0.695 | 0.652 | 0.638 | 0.763 | 0.704 |

|  | *Phalanger* | *Ailurops* | *Spilocuscus* | *Vombatus* | *P. nasuta* | *P. gunnii* |
| --- | --- | --- | --- | --- | --- | --- |
|  |  |  |  |  |  |  |
| *D. hallucatus* | 0.625 | 0.319 | 0.733 | 0.496 | 0.245 | 0.656 |
| *D. maculatus* | 0.573 | 0.240 | 0.752 | 0.500 | 0.190 | 0.570 |
| *D. viverrinus* | 0.673 | 0.260 | 0.782 | 0.458 | 0.206 | 0.694 |
| *Phascogale* | 0.621 | 0.316 | 0.691 | 0.446 | 0.236 | 0.659 |
| *Sarcophilus* | 0.570 | 0.290 | 0.779 | 0.526 | 0.209 | 0.636 |
| *Dasyuroides* | 0.598 | 0.308 | 0.702 | 0.464 | 0.243 | 0.670 |
| *Myrmecobius* | 0.629 | 0.603 | 0.392 | 0.712 | 0.670 | 0.444 |
| *Thylacinus* | 0.543 | 0.458 | 0.710 | 0.601 | 0.316 | 0.607 |
| *Trichosurus* | 0.576 | 0.708 | 0.377 | 0.652 | 0.665 | 0.347 |
| *Phalanger* | 1.000 | 0.438 | 0.637 | 0.618 | 0.502 | 0.557 |
| *Ailurops* | 0.867 | 1.000 | 0.299 | 0.708 | 0.692 | 0.281 |
| *Spilocuscus* | 0.780 | 0.767 | 1.000 | 0.489 | 0.195 | 0.652 |
| *Vombatus* | 0.672 | 0.688 | 0.781 | 1.000 | 0.718 | 0.410 |
| *P. nasuta* | 0.675 | 0.734 | 0.798 | 0.731 | 1.000 | 0.215 |
| *P. gunnii* | 0.562 | 0.613 | 0.806 | 0.732 | 0.877 | 1.000 |
| *Isoodon* | 0.712 | 0.706 | 0.892 | 0.725 | 0.850 | 0.848 |
| *Macrotis* | 0.725 | 0.721 | 0.895 | 0.744 | 0.877 | 0.797 |
| *Echymipera* | 0.656 | 0.679 | 0.824 | 0.731 | 0.913 | 0.873 |
| *Microperoryctes* | 0.557 | 0.607 | 0.743 | 0.728 | 0.854 | 0.857 |
| *Peroryctes* | 0.538 | 0.548 | 0.751 | 0.675 | 0.777 | 0.833 |

|  | *Isoodon* | *Macrotis* | *Echymipera* | *Microperoryctes* | *Peroryctes* |
| --- | --- | --- | --- | --- | --- |
|  |  |  |  |  |  |
| *D. hallucatus* | 0.677 | 0.605 | 0.664 | 0.630 | 0.413 |
| *D. maculatus* | 0.672 | 0.572 | 0.629 | 0.485 | 0.359 |
| *D. viverrinus* | 0.730 | 0.599 | 0.692 | 0.573 | 0.353 |
| *Phascogale* | 0.663 | 0.566 | 0.609 | 0.628 | 0.438 |
| *Sarcophilus* | 0.707 | 0.631 | 0.687 | 0.596 | 0.364 |
| *Dasyuroides* | 0.691 | 0.630 | 0.679 | 0.660 | 0.407 |
| *Myrmecobius* | 0.388 | 0.569 | 0.402 | 0.433 | 0.623 |
| *Thylacinus* | 0.657 | 0.748 | 0.641 | 0.602 | 0.392 |
| *Trichosurus* | 0.310 | 0.584 | 0.340 | 0.343 | 0.549 |
| *Phalanger* | 0.551 | 0.599 | 0.547 | 0.518 | 0.590 |
| *Ailurops* | 0.309 | 0.584 | 0.337 | 0.355 | 0.574 |
| *Spilocuscus* | 0.717 | 0.644 | 0.645 | 0.583 | 0.366 |
| *Vombatus* | 0.456 | 0.661 | 0.427 | 0.415 | 0.717 |
| *P. nasuta* | 0.214 | 0.481 | 0.219 | 0.251 | 0.809 |
| *P. gunnii* | 0.656 | 0.549 | 0.682 | 0.692 | 0.311 |
| *Isoodon* | 1.000 | 0.647 | 0.689 | 0.575 | 0.381 |
| *Macrotis* | 0.925 | 1.000 | 0.581 | 0.558 | 0.518 |
| *Echymipera* | 0.860 | 0.858 | 1.000 | 0.671 | 0.322 |
| *Microperoryctes* | 0.789 | 0.748 | 0.876 | 1.000 | 0.328 |
| *Peroryctes* | 0.774 | 0.730 | 0.883 | 0.831 | 1.000 |
